# Etiology of precocious puberty, 10 years study in Endocrine Reserch Centre (Firouzgar), Tehran

**Published:** 2012-01

**Authors:** Farzaneh Rohani, Shadab Salehpur, Fatemeh Saffari

**Affiliations:** 1 Endocrine Research Centre (Firouzgar), Institute of Endocrinology and Metabolism (Hemmat Campus), Tehran University of Medical Sciences, Tehran, Iran.; 2 Ali Asghar Children’s Hospital, Tehran University of Medical Sciences, Tehran, Iran.; 3 Mofid Children’s Hospital, Shahid Beheshti University of Medical Sciences, Tehran, Iran.; 4 Booali Metabolic Research Center, Qazvin University of Medical Sciences, Qazvin, Iran.

**Keywords:** *Central precocious puberty*, *Peripheral precocious puberty*, *Premature thelarche*, *Congenital adrenal hyperplasia*

## Abstract

**Background:** Precocious puberty, as early physical development and low final height might lead to psychosocial problems.

**Objective:** To evaluate etiology and clinical feature of precocious puberty in a cohort of Iranian children.

**Materials and Methods:** In this case-series study, 44 girls and 8 boys with precocious puberty referred to Endocrine Reserch Centre (Firouzgar), Institute of Endocrinology and Metabolism (Hemmat Campus), were examined in a 10 years period of time.

**Results:** Mean age of girls and boys was 7.43±1.4 years and 5.8±2.1 years respectively. Most of the patients fell within the age category of 7-7.9 years old (40.9% for girls and 50% for boys). Patients, concerning etiology of precocious puberty were classified in three categories: 42.6% of patients had central precocious puberty (CPP), including idiopathic CPP (87.5%) and neurogenic CPP (12.5%). 23.3% of patients had peripheral precocious puberty (PPP), including congenital adrenal hyperplasia (CAH) (42.8%), ovarian cysts (28.4%), McCune-Albright syndrome (14.2%) and adrenal carcinoma (14.2%). 34.1% of girls and 25% of boys had normal variant puberty including premature thelarche (57%), premature adrenarche (38%) as well as premature menarche (4.7%l).

**Conclusion: **The most common etiology of precocious puberty in girls was idiopathic central precocious puberty and premature thelarche, while in boys they were neurogenic central precocious puberty and CAH. Therefore precocious puberty in girls is usually benign. In boys, CNS anomalies should first be considered in the differential diagnosis of CPP. Therefore brain Magnetic Resonance Imaging (MRI) is mandatory in all cases.

## Introduction

Precocious precocity is defined as the appearance of secondary sexual characteristics in an age that is less than 2-2.5 standard deviation below the mean age of puberty for general population (1, 2) or the onset of menstruation before 9.5 year-old in girls (3). 

Most of endocrinologist consider the age of 8 in girls and 9 in boys as the lowest age of normal puberty (4-12), Precocious puberty can occur because of a disturbance in hypothalamic pituitary gonadal axis (which is called central precocious puberty), or because of a disturbance outside that axis (which is called peripheral precocious puberty), or may be extreme variations of normal (normal variant). 

Central precocious puberty (CPP) may be a consequence of a known or an identifiable underlying central nervous system disturbance (neurogenic central precocious puberty: NCPP) or may be associated with no apparent abnormality (idiopathic central precocious puberty: ICPP). 

The cause of NCPP is central nervous system irradiation, brain tumor, head trauma, and brain chronic inflammatory disorders. Peripheral precocious puberty (PPP) results from androgen or estrogen excess independent of hypothalamic pituitary-gonadal activity. The cause of PPP is ovarian cyst or tumor, adrenal tumor, congenital adrenal hyperplasia (CAH), and McCune-Albright syndrome. Partial pubertal development unassociated with other significant pubertal change has been considered as variations of normal (normal variant). 

Normal variant include isolated breast development (premature thelarche), isolated pubic hair appearance (premature adrenarche) and isolated vaginal bleeding (premature menarche) (3). In addition to evolution of secondary sexual characteristics, the puberty is associated with an accelerated rate of growth and bone maturation. In patients with precocious puberty early exposure to sex steroids (estrogen or testosterone) lead to premature skeletal maturation and consequently premature fusion of epiphysial growth plate (13). This can lead to an overall decrease in adult height (14, 15). Early physical development and short adult height would in turn lead to numerous socio- psychic problems. In spite of the importance and relative prevalence of precocious puberty, limited data on etiology of precocious puberty in the Iranian patients is available (16, 17). 

The objective of this study is to examine the etiology and clinical features of precocious puberty in cohort of Iranian children. The study supports required planning to raise awareness of physicians for early diagnosis and treatment of the diseases.

## Materials and methods

This case-series study was conducted in the Endocrine Reserch Centre (Firouzgar), Institute of Endocrinology and Metabolism (Hemmat Campus), affiliated to the Tehran University of Medical Science (TUMS). 

Patients with precarious puberty (appearance of secondary sexual characteristics in girls younger than 8 years old and in boys younger than 9 years old), referred to the Endocrine Reserch Centre (Firouzgar), Institute of Endocrinology and Metabolism (Hemmat Campus), in a 10 years period, were enrolled in the study through non-probability convenience sampling method. 

Data on sex, age, height, weight, skeletal age (in accordance with the Greulich and Pyle Atlas) (18), disease background, puberty stage (in accordance with the Tanner category) (19, 20), baseline LH and FSH levels, and if required FSH and LH levels after GnRH stimulation test (GnRH: 0.1 mg/m²) (21), estradiol, testosterone, thyroxin, TSH, adrenal androgens, pelvic ultra sonography and computed tomography (CT) or magnetic resonance imaging (MRI) of the brain were extracted from the patients records and categorized in data sheets. 

**Statistical analysis**

Statistical analysis was performed by SPSS 13 software. The results are reported as mean±SD.

## Results

Out of 52 patients with precocious puberty, 86.4% were girls (44 girls), and 15.4% were boys (8 boys). Mean age for girls and boys under the study was 7.43±1.4 and 5.8±2.1 years respectively. Most patients fell under the age category of 7-7.9 years old (40.9% of girls and 50% of boys). According to the clinical examinations and para clinical findings, patients in terms of precocious puberty etiology were classified in three categories of CPP, PPP and normal variant puberty. 

**Figure 1:**
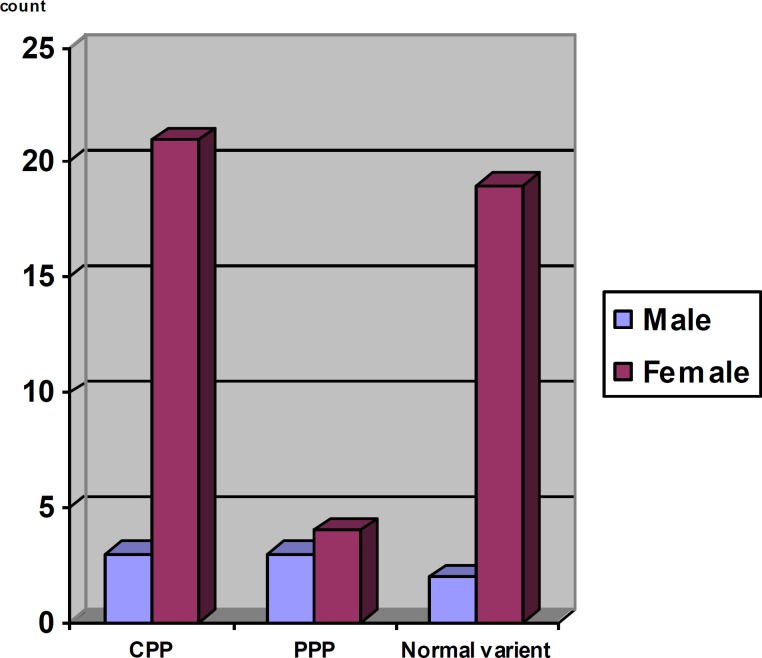
Frequency of etiology of precocious puberty with sex distinction.

The most common type of precocious puberty in girls was CPP (47.7%) and in boys was CPP (37.5%) and congenital adrenal hyperplasia (37.5%). The frequency of each category, with sex distinction is demonstrated in Figure 1. CPP was identified in 21 girls (47.7%) and 3 boys (37.5%). Out of the above, 20 girls and 1 boy diagnosed with idiopathic central precocious puberty and 1 girl and 2 boys diagnosed with neurogenic central precocious puberty. PPP was identified as the etiology of 9.1% (4 girls) and 37.5% (3 boys) of precocious puberty in girls and boys respectively. Ovarian cysts in 2 girls, McCune-Albright syndrome in 1 girl and adrenal carcinoma in 1 girl were identified as the etiology of peripheral precocious puberty. 

**Figure 2:**
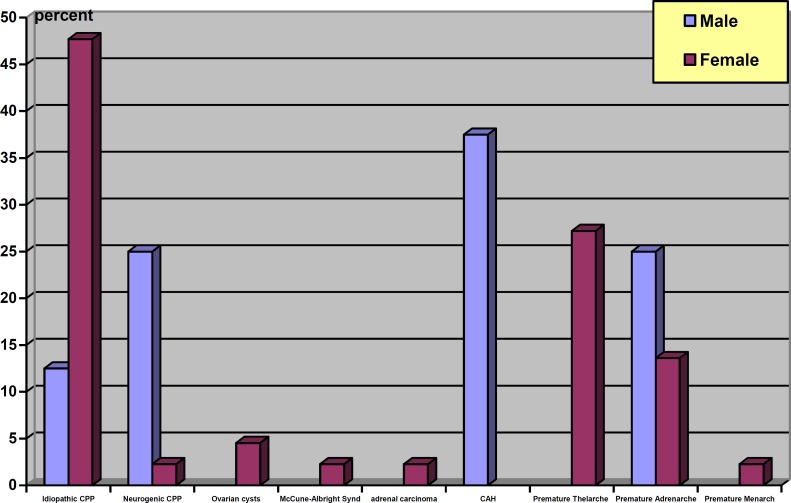
Frequency percentile of etiology of precocious puberty in male and female

All 3 boys with peripheral precocious puberty were diagnosed with congenital adrenal hyperplasia. Normal variant puberty was the etiology of 43.2% of precocious puberty in girls and 25% of boys. Out of 19 girls in this category, 12 girls were diagnosed with premature thelarche, 6 patients with premature adrenarche and 1 girl with premature menarche. The etiology behind precocious puberty in 2 boys under this category was premature adrenarche. Frequency percentile of precocious puberty etiology is displayed in Figure 2.

**Table I:** Sex incidence of precocious puberty in different studies

**Investigator **	**Patient number**	**Female**	**Male**	**Female to male ratio**
Brauner (1982) (23)	124	92	32	2.9: 1
Bridges (1994) (24)	213	197	16	12.3: 1
Chemaitilly (2001) (25)	256	230	26	8.9: 1
Bajpai (2002) (26)	140	114	26	4.4: 1
Moayery (2002) (17)	75	50	24	2: 1
Soriano-Guillen (2010) (24)	250	226	24	9.4: 1
Rohani (2010)	52	44	8	5.5: 1

**Table II:** Sex incidence of idiopathic and neurogenic precocious puberty in different studies

**Investigator **	**ICPP**		**NCPP**			
	**Male**	**Female**	**Male**	**Female**		
Thamdrup (1961) (29)	4	34	7	11	3: 1	1: 0.57
Wklkins (1965) (30)	13	67	10	5	13: 4	1.3: 1
Sigurjonsdottir & Hayle (1968) (31)	8	54	16	16	3: 1	1: 2
University of California (1981) (32)	13	121	26	45	7: 2	1: 2
Bridges (1994) (24)	0	85	3	6	14: 1	0
Cisternino (2000) a (33)	-	226	-	56	4: 1	-
De Sanctis(2000) b (28)	27	-	18	-	-	1: 1.5
Chematilly (2001) (25)	7	186	19	44	4: 2	0.37: 1
Bajpai (2002) (26)	8	61	10	16	8: 3	1: 0.8
Ghaemi (2002) a (16)	-	34	-	4	8.5: 1	-
Lee (2009) (34)	-	427	-	17	25: 1	-
Rohani (2010)	1	20	2	1	20: 1	1: 2

ICPP: Idiopathic Central precocious Puberty.

NCPP: Neurogenic Central precocious puberty.

a . Study only in female.

b . Study only in male.

c . ICPP to NCPP ratio in female.

d . ICPP to NCPP ratio in male.

**Table III:** Frequency of PPP and normal variant in girls, in some studies

**Investigator**	**Bridges (21)**	**Cisternin (32)**	**Bajpai (23)**	**ZOU CC (35)**	**Ghaemi (16)**	** Moayeri (17)**	**Rohani**
Etiology of P.P	% total patients with P.P	% total patients with P.P	% total patients with P.P	% total patients with P.P	% total patients with P.P	% total patients with P.P	% total patients with P.P
Ovarian cyst	-	0.23%	-	37%	27.7%	-	4.54%
Ovarian hyper functioning	-	0.68%	-	-	-	-	-
Ovarian tumor	-	0.68%	-	1.5%	-	-	-
McCune-Albright syndrome	-	0.68%	0.88%	4.6%	-	2%	2.27%
Adrenal hyperplasia	-	-	-	15%	18.2%	-	-
Adrenal carcinoma	-	-	-	-	18.2%	-	2.27%
Hypothyroidism	0.5%	-	3.5%	-	27.7%	2%	-
Premature thelarche	-26%	-	20.24%	-	-	46%	27.2%
Premature adrenarche	-10%	-	5.24%	-	-	8%	13.62%
Premature menarche	-	-	-	-	-	2%	2.27%
Thelarche variant	-15%	-	-	-	-	-	-
Total number of patients with P.P	(n=197) 100%	(n=438) 100%	(n=114) 100%	(n=65) 100%	(n=55) 100%	(n=65) 100%	(n=44) 100%

a. CCP. Central precocious puberty.

b. PPP. Peripheral precocious puberty.

CPP: Central Precocious Puberty.

CAH: Congenital Adrenal Hyperplasia.

## Discussion

Incidence of precocious puberty is estimated to be 1 per 5000-10,000 individuals. In spite of the importance and relative prevalence of precocious puberty, to our knowledge, there is limited data on etiology of precocious puberty in the Iranian patients (16, 17). The Ghaemi *et al* (16) undertook a review of 55 girls in Mashhad and another study was also conducted by Moayeri *et al* (17) on 74 patients with precocious puberty in Imam Hospital, in Tehran.

Precocious puberty is present more commonly in girls than boys (female to male ratio 5-10:1 approximately). (15-22) In our study 86.4% of patients were girls and 15% were boys, therefore female to male ratio was 5.5:1. 

This is consistent with the conclusions of the endocrinology text book. In studies by Brauner *et al* (23) (Paris), Bridges *et al* (24) (London), Chemaitilly *et al* (25) (Paris), Bajpai *et al* (26) (Delhi), Soriano-Guillen *et al* (27) (Spain), and Moayeri *et al* (17) (Tehran), 74%, 92%, 89%, 81%, 90%, 67.6% of patients were girls respectively. Sex incidence of precocious puberty in different studies is summarized in table I, an indication of the female to male ratio of 5-10 to 1. Our data demonstrate that CPP presents more commonly in girls (47.7% of girls) than boys (37.5% of boys). 

The majority of girls with CPP (95%) had Idiopathic CPP and no cause was found. (ICPP to NCPP ratio, 20:1). Whereas a neurogenic cause was identified in 66.6% of boys (ICPP to NCPP ratio, 1:2)

In Moayeri *et al* study, 40% of female patients had CPP, 90% of which were idiopathic CPP. Furthermore, 38.8% of boys with CPP had neurogenic cause while 61% of them had ICPP. In another study from Italy (28), on 45 boys with CPP, 60% of them had ICPP while the remaining 40% had neurogenic CPP. 

Contrary to our findings and Endocrine references, in these two studies, ICPP in boys was more common than NCPP. Sex incidence of precocious puberty, with the distinction of idiopathic and neurogenic precocious puberty in different studies is demonstrated in table II. 

Findings of the most studies reiterate the incidence of idiopathic central precocious puberty in girls and neurogenic central precocious puberty in boys. The etiology of idiopathic precocious puberty in girls is unknown. This conclusion, however, is due to the assumption of relatively simple reactivation of hypothalamic-pituitary- gonadal axis in girls in comparison to boys (15). The incidence of neurogenic central precocious puberty in boys requires more careful examination of central precocious puberty as well as conducting computed tomography or magnetic resonance imaging of the brain in all patients.

According to the present study, 9.1% of girls and 37.5% of boys had PPP, while all of them (100%) were affected by CAH. In Moayeri *et al* (6 boys with PPP) and ZOU *et al* studies (35) (26 boys with PPP), CAH was identified as the most common cause of PPP. These results imply that CAH is a common matter in boys with PPP.

Normal variant puberty is isolated manifestation of precocious puberty without any other evidence of puberty. In our study normal variant was considered as the second common cause (43.2%) of puberty in girls with dominancy of premature thelarche. Moayeri *et al* study showed 54% of girls with precocious puberty, had normal variant and 46% of them had premature thelarche.

In accordance with our finding, supported by the conclusions of Bridges *et al* (24) and Bajpai *et al* (26) studies, normal variant was the cause of precocious puberty in 51% and 25.5% of patients respectively. 

Moreover, premature thelarche was identified as the most common sub group of normal variant in these studies. Additionally, premature thelarche is a benign condition (36) which dose not require treatment. Frequency of PPP and normal variant etiology in girls, in some studies are demonstrated in table III.

## Conclusion

The most common etiology of precocious puberty in girls was ICPP (47.7%) and premature thelarche (34.1%). Therefore dealing with a girl with precocious puberty, requires consideration of ICPP as well as premature thelarche, as the first diagnosis.

The most common etiology of precocious puberty in boys was NCPP (25%) and CAH (37.5%). In boys who refer due to CPP or PPP, NCPP in the first group and CAH in the second group, should be taken into consideration as the first diagnosis. According to our study as supported by other references, precocious puberty in girls is usually benign but in boys it should be considered and evaluated seriously. This is because when CPP occurs in boys, it is more likely to be the result of a demonstrable CNS lesion. Therefore, in boys with CPP, conducting neuro radiological imaging (either CT or MRL) is mandatory.
